# Acute effects of insulin on circulating natriuretic peptide levels in humans

**DOI:** 10.1371/journal.pone.0196869

**Published:** 2018-05-14

**Authors:** Katherine N. Bachmann, Serpil Muge Deger, Aseel Alsouqi, Shi Huang, Meng Xu, Jane F. Ferguson, Yan Ru Su, Kevin D. Niswender, T. Alp Ikizler, Thomas J. Wang

**Affiliations:** 1 Veterans Administration Tennessee Valley Healthcare System, Nashville, Tennessee, United States of America; 2 Division of Diabetes, Endocrinology and Metabolism, Department of Medicine, Vanderbilt University Medical Center, Nashville, Tennessee, United States of America; 3 Vanderbilt Translational and Clinical Cardiovascular Research Center, Vanderbilt University School of Medicine, Nashville, Tennessee, United States of America; 4 Division of Nephrology, Department of Medicine, Vanderbilt University Medical Center, Nashville, Tennessee, United States of America; 5 Department of Biostatistics, Vanderbilt University Medical Center, Nashville, Tennessee, United States of America; 6 Division of Cardiovascular Medicine, Department of Medicine, Vanderbilt University Medical Center, Nashville, Tennessee, United States of America; 7 Department of Molecular Physiology and Biophysics, Vanderbilt University School of Medicine, Nashville, Tennessee, United States of America; 8 Vanderbilt Center for Kidney Disease, Vanderbilt University Medical Center, Nashville, Tennessee, United States of America; Ospedale del Cuore G Pasquinucci Fondazione Toscana Gabriele Monasterio di Massa, ITALY

## Abstract

**Background:**

The natriuretic peptide hormones play an important role in salt and blood pressure regulation. In observational studies, obesity and insulin resistance have been consistently associated with lower concentrations of natriuretic peptides. It has been proposed that insulin influences natriuretic peptide production.

**Objective:**

We sought to determine the acute effects of insulin administration on natriuretic peptide concentrations.

**Methods:**

31 men and women (11 lean, 10 overweight, and 10 obese), ages 30–70 years, without cardiovascular disease or overt diabetes underwent a hyperinsulinemic-euglycemic insulin clamp. Plasma concentrations of N-terminal pro atrial natriuretic peptide (NT-proANP) and N-terminal pro B-type natriuretic peptide (NT-proBNP) were measured at baseline and steady-state (the final 30 minutes of the clamp protocol).

**Results:**

From baseline to steady-state, insulin levels increased from a mean level of 9.5 to 176.7 μU/ml (*p*<0.001). Over this period, circulating NT-proANP concentrations decreased by 9% (-1933 ng/L, p = 0.01). The changes in NT-proANP did not differ between lean, overweight, and obese individuals. Steady-state NT-proANP levels, adjusted for baseline, were lower in individuals with greater insulin resistance, independent of BMI. In contrast to NT-proANP, NT-proBNP levels did not change significantly during the clamp (p = 0.41).

**Conclusion:**

Insulin administration was associated with a moderate decrease in circulating NT-proANP, but not NT-proBNP. The lowest NT-proANP concentrations were found in insulin-resistant individuals. Further investigations are warranted to elucidate potential mechanisms underlying the effects of insulin on the cardiac hormonal axis.

## Introduction

The cardiac natriuretic peptide (NP) hormonal system plays an important role in salt balance, blood pressure regulation, and cardiovascular homeostasis. Natriuretic peptides appear to protect against the development of hypertension and cardiovascular risk [1–3]. In addition, emerging evidence suggests that the NP system may play a protective role in metabolism [4–7].

Large cohort studies have demonstrated that obese individuals have lower circulating levels of both atrial natriuretic peptide (ANP) and B-type natriuretic peptide (BNP), compared with lean individuals [8, 9]. The low NP levels in obese individuals appear to reflect a relative “NP deficiency.” Given the protective role of the NPs, a relative NP deficiency in obesity may contribute to the greater risk of cardiometabolic disease in obese individuals.

The mechanisms underlying the low NP levels in obesity are not well-understood. Proposed explanations include increased clearance of NPs in adipose tissue in obese subjects, secretion of bioactive substances by adipose tissue that affect NP production in the heart, alterations in sex hormone production and activity, and insulin resistance [10]. Regarding the latter hypothesis, some evidence suggests that insulin resistance contributes to lower NP levels, above and beyond adiposity [9]. Thus, NP levels in individuals who are lean but insulin-resistant are nearly as low as those in obese, insulin-resistant individuals [9]. These observations raise the possibility that insulin itself may influence NP production, but data from human studies are mixed [11–21]. The hyperinsulinemic-euglycemic insulin clamp is a useful technique that permits the effects of insulin to be examined, independent of excursions in glucose. Thus, we performed a physiologic study using this technique to examine the acute effects of high-dose insulin administration on circulating NP levels.

## Materials and methods

### Subjects

This study included 31 men and women: 11 lean (BMI < 25 kg/m^2^), 10 overweight (25 ≤ BMI < 30 kg/m^2^), and 10 obese (BMI ≥ 30 kg/m^2^). Subjects were between 30 and 70 years of age (mean age, 50 years) and had a history of pre-hypertension, as defined by systolic blood pressure 120–139 mmHg and/or diastolic blood pressure 80–89 mmHg. Subjects with a history of pre-hypertension were recruited because this study was part of a larger study focused on the development of hypertension among individuals with pre-hypertension. All study procedures in the present report were performed at the baseline visit prior to any intervention. Exclusion criteria were cardiovascular events within the prior 6 months, impaired renal function (estimated glomerular filtration rate < 45 ml/min/1.73m^2^), impaired hepatic function (aspartate amino transaminase and/or alanine amino transaminase > 1.5 times the upper limit of normal range), diabetes mellitus requiring medical therapy, morbid obesity (BMI > 45 kg/m^2^), current use of anti-hypertensive medication or glucocorticoids, and pregnancy. Cardiovascular history was assessed by conducting a thorough history from the individual at the screening visit. None of the subjects in the present report had experienced an acute cardiovascular event in the prior 6 months, nor had a history of coronary artery disease, myocardial infarction, congestive heart failure, pacemaker/defibrillator, peripheral vascular disease, or abnormalities on cardiovascular physical exam. We did not perform formal echocardiographic studies to exclude cardiac disease in these subjects. A complete metabolic panel and a urine sample were collected to rule out kidney and hepatic dysfunction. The Institutional Review Board at Vanderbilt University Medical Center approved the study protocol, and written informed consent was obtained from all subjects.

### Study design

Study procedures were performed at the General Clinical Research Center at Vanderbilt University Medical Center. Data included in the present report were collected during 11/2014–1/2016. After fasting overnight for at least 8 hours, subjects underwent a hyperinsulinemic-euglycemic insulin clamp protocol, using a primed continuous intravenous infusion of human insulin at the rate of 2 mU/kg/min throughout the clamp protocol. Plasma glucose concentration was measured at the bedside every 5 minutes, and an infusion of 20% dextrose was titrated to maintain the basal blood glucose level. After steady-state conditions were achieved (target glucose maintained with a constant dextrose infusion rate), the clamp was continued for a final 30 minutes. The final 30 minutes of the clamp were considered the steady-state period. The duration of the clamp protocols ranged from approximately 100–150 minutes, depending on the time required to achieve steady-state conditions. The total body glucose disposal rate (GDR, mg/kg/min) was determined using the glucose infusion rate during the steady-state period, and served as a measure of *in vivo* insulin sensitivity.

### Hormone measurements

Plasma N-terminal pro atrial natriuretic peptide (NT-proANP) (1–98) concentration was measured using a sandwich ELISA assay (Biomedica, Vienna, Austria). All samples in the present study were run in duplicate, with an average CV of < 7%. Plasma N-terminal pro B-type natriuretic peptide (NT-proBNP) concentration was measured using an electrochemiluminescence immunoassay (Roche Elecsys ProBNP II, Roche Diagnostics, Indianapolis, IN). Details of this method have been reported previously [22]. The intra-assay CV is ≤ 1.2% at the Vanderbilt research laboratory. NP levels were measured at baseline, and at the beginning and end of the 30-minute steady-state period. The “steady-state” value refers to the mean of the values at the beginning and end of steady-state.

Plasma insulin concentration was determined by radioimmunoassay using a double antibody procedure (MilliporeSigma, Billerica, Massachusetts). The inter-assay and intra-assay CVs are 9.9% and 2.9%, respectively, at the Vanderbilt research laboratory that tested the samples in the present study. Plasma glucose concentration was measured by the glucose oxidase method (Analox GM9 glucose analyser; Analox Instruments, Stourbridge, United Kingdom).

Blood samples were drawn into tubes containing EDTA (K3) 0.10 mL 15% solution tube (Covidien) and were placed on ice immediately. Within 10 minutes on average, the samples were centrifuged at 3000 rpm for 15 minutes. They were transferred to aliquot tubes, and stored at -80°C until testing in batches at Vanderbilt research laboratories. No freeze-thaw cycles occurred prior to testing. Samples were stored at -80°C for ≤16 months. <24 months, and ≤15 months before testing for NT-proANP, NT-proBNP, and insulin levels, respectively.

### Statistical analysis

**Fig 1:**
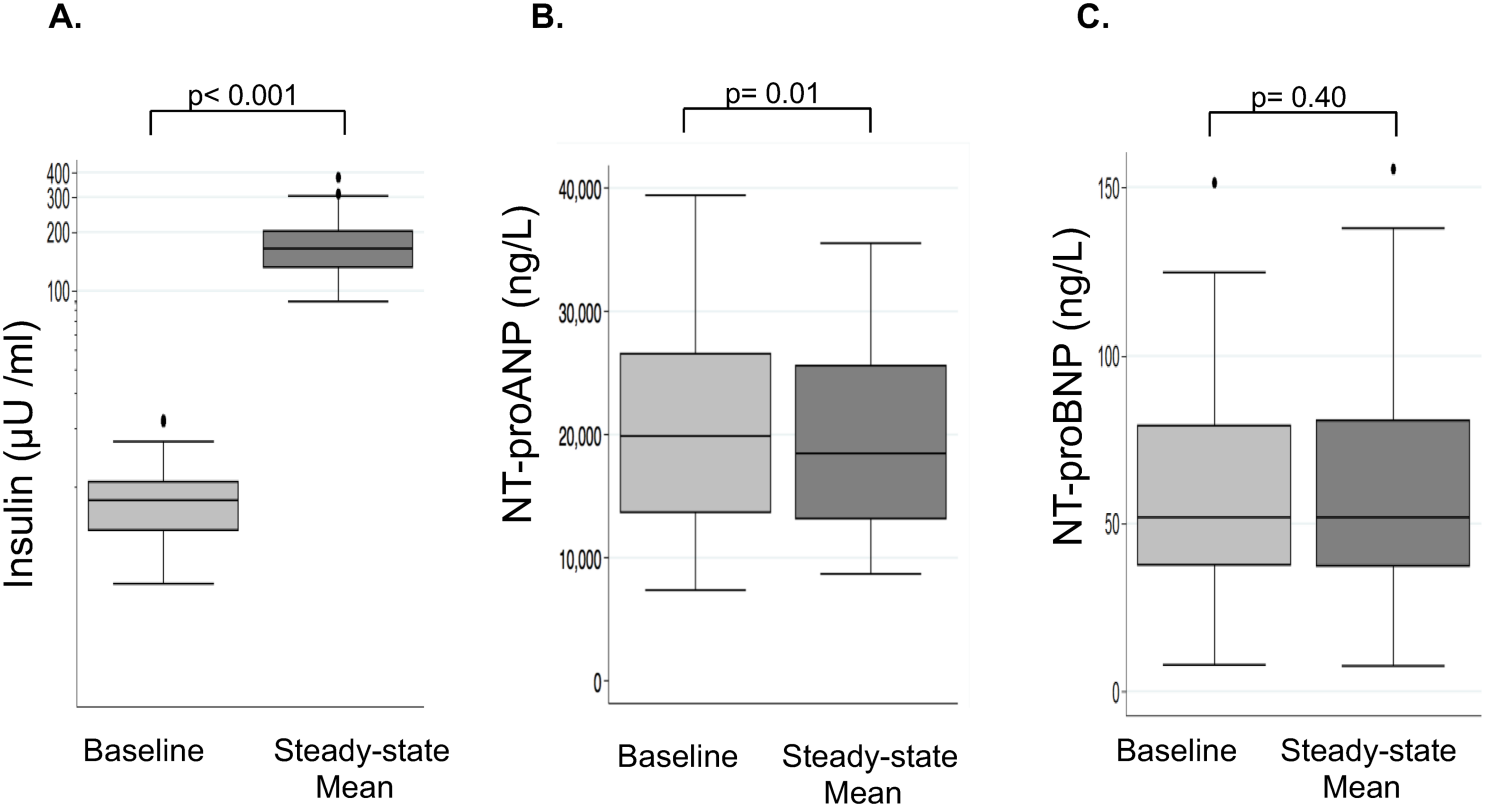
Circulating insulin and NP concentrations during hyperinsulinemic-euglycemic clamp. *(Panel A)* Insulin levels increased from baseline to steady-state (mean increase of 167 μU/ml, *p*<0.001) in the group as a whole. *(Panel B)* NT-proANP in the group as a whole decreased from baseline to steady-state (mean decrease of 1933 ng/L, p = 0.01), representing a 9% relative decrease. *(Panel C)* NT-proBNP did not change significantly from baseline to steady-state in the group as a whole (mean change -0.45 ng/L, p = 0.40).

Baseline characteristics were reported as median (lower quartile, upper quartile) for continuous variables and as percentages for categorical variables. Descriptive statistics at baseline were compared between the 3 BMI categories (lean, overweight, and obese) using the Kruskal-Wallis test for continuous variables and the Pearson Chi-square test for categorical variables. Insulin and natriuretic peptide concentrations (Fig 1) were reported in box plots displaying the median, interquartile range, and upper and lower adjacent values.

Baseline and steady-state NP levels were compared using the paired Wilcoxon signed rank test. Next, we examined which factors impacted the changes in NP levels using multivariable regression. Changes in NP levels were analyzed using multivariable linear regression, with steady-state NP level as the response variable, and with baseline NP level as an independent variable (e.g. “steady-state NP adjusted for baseline”), which is equivalent to NP changes. In order to examine which factors impacted the changes in NP levels, we included pre-specified predictors, including BMI, age, sex, race (white vs. non-white), and glucose disposal rate, as independent variables in the regression. Two-sided p-values <0.05 were considered statistically significant. R version 3.3.1 (R foundation of Statistical Computing, Vienna, Austria) was used for statistical analyses.

## Results

Baseline characteristics of the subjects, stratified by BMI category are displayed in Table 1. Baseline insulin levels were slightly higher in obese individuals compared with lean and overweight individuals, but the magnitude of the differences was small. Glucose levels did not differ at baseline. Glucose disposal rate during steady-state (a measure of insulin sensitivity) was significantly lower in obese individuals compared with lean (p = 0.02) and overweight (p = 0.02) individuals.

**Table 1:** Baseline characteristics.

	Lean	Overweight (OW)	Obese (OB)	*p-value*			
	(N = 11)	(N = 10)	(N = 10)	Overall	Lean vs. OW	Lean vs. OB	OW vs. OB
Age (years)	54 (47, 60)	48.5 (41, 61)	50.5 (40, 58)	NS	-	-	-
Sex (% male)	36.4%	30.0%	0%	0.039			
Race (% white)	63.6%	80.0%	60.0%	NS	-	-	-
BMI (kg/m^2^)	23.6 (22.5, 24.8)	28.5 (26.5, 29.2)	34.5 (31.2, 41.8)	<0.0001	0.0001	0.0001	0.0002
Height (cm)	167 (158, 172)	169 (165, 174)	166 (162, 169)	NS	-	-	-
Weight (kg)	63.1 (61.9, 71.2)	81.3 (72.4, 85)	96.4 (84, 115.2)	<0.0001	0.0004	0.0001	0.0140
Systolic BP (mm Hg)	135 (129, 142)	128 (121, 129)	132.5 (127, 146)	NS	-	-	-
Diastolic BP (mmHg)	80 (74, 90)	74 (70, 86)	79.5 (71, 81)	NS	-	-	-
Heart rate (beats/minute)	67 (61, 69)	63 (52, 70)	66 (65, 73)	NS	-	-	-
Insulin (μU/ml), Min 0	8.0 (4.2, 9.0)	7.8 (5.9, 9.1)	10.0 (8.8, 13.0)	0.049	NS	0.032	0.045
Glucose (mg/dl), Min 0	93 (86, 102)	99.5 (94, 104)	92.5 (87, 98)	NS			
Glucose disposal rate (mg/kg/min)	10.8 (6.1, 14.0)	10.8 (6.7, 12.4)	6.4 (5.2, 8.1)	0.029	NS	0.024	0.019
NT-proANP (ng/L), Min 0	21873 (13709, 26873)	24082 (13304, 31861)	19253 (13924, 24734)	NS	-	-	-
NT-proBNP (ng/L), Min 0	49.7 (34.9, 73.7)	43.8 (36.1, 79.3)	62.1 (43.9, 84.5)	NS	-	-	-

Lean group had BMI< 18.5 kg/m^2^. Overweight group had 18.5≤BMI<25 kg/m^2^. Obese group had BMI> 30 kg/m^2^. Results for continuous variables are reported as medians (25^th^ percentile, 75^th^ percentile). OW, overweight; OB, obese; NS, not significant; BMI, body mass index; BP, blood pressure; NT-proANP, N-terminal pro atrial natriuretic peptide; NT-proBNP, N-terminal pro B-type natriuretic peptide

Circulating baseline and steady-state (the mean of the values at the beginning and end of steady-state) concentrations of insulin, NT-proANP, and NT-proBNP are shown in Fig 1A, 1B and 1C, respectively. As expected, insulin levels increased (from a mean level of 9.5 μU/ml to 176.7 μU/ml, p<0.001) during the hyperinsulinemic-euglycemic clamp (Fig 1A).

Circulating NT-proANP levels decreased from baseline to the mean of steady-state levels (mean decrease of 1933 ng/L, 95% confidence interval [557–3309], p = 0.01), representing a 9% relative decrease, in the group as a whole (Fig 1B). The magnitude of changes in NT-proANP levels did not differ according to BMI category (p = 0.38) or continuous BMI (p = 0.68). In contrast to NT-proANP, circulating NT-proBNP levels did not change significantly from baseline to steady-state in the group as a whole (mean change -0.45 ng/L, p = 0.40) (Fig 1C). Results were very similar when analyzing changes from baseline to the beginning of steady-state, and from baseline to the end of steady-state (S1 Fig). Furthermore, results were similar in secondary analyses restricted to women.

**Fig 2:**
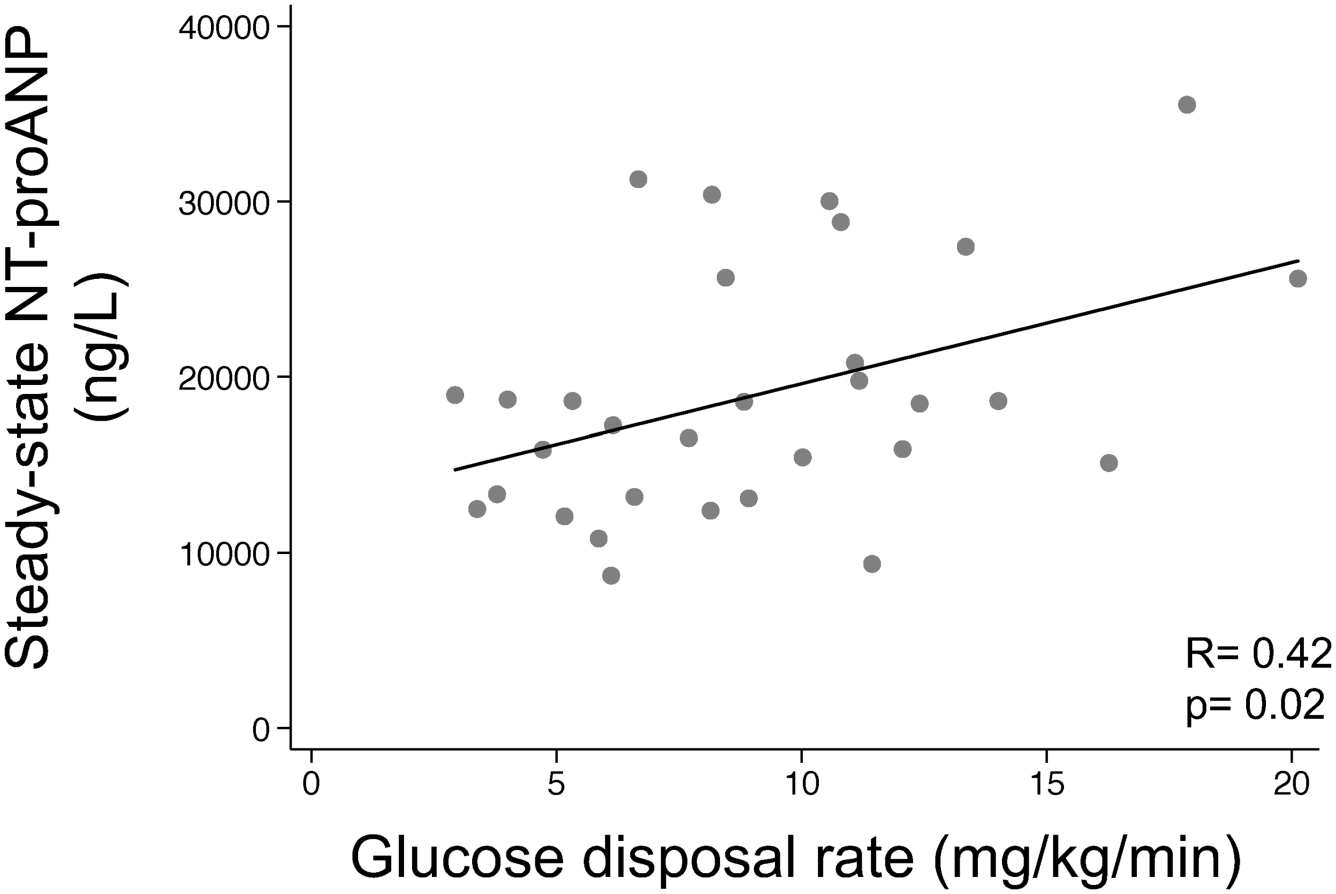
Relationship of changes in NT-proANP level and glucose disposal rate. Steady-state NT-proANP levels were positively associated with glucose disposal rate (a measure of insulin sensitivity). This relationship persisted after adjustment for baseline NT-proANP levels, BMI, age, sex, and race.

Steady-state NT-proANP levels were positively associated with glucose disposal rate (R = 0.42, p = 0.02) (Fig 2). Moreover, changes in NT-proANP levels (steady-state adjusted for baseline) were positively associated with glucose disposal rate, both in unadjusted models (partial R = 0.50, p = 0.005) and after adjustment for BMI, age, sex, and race (partial R = 0.49, p = 0.01). A lower glucose disposal rate by 1 mg/kg/min was associated with a lower steady-state NT-proANP level by 456 ng/L, after adjustment for baseline NT-proANP, BMI, age, sex, and race. We also tested interaction terms between BMI subgroup and glucose disposal rate and found that the interactions were not significant (p = 0.29 for NT-proANP by BMI subgroup, and p = 0.74 for NT-proBNP by BMI subgroup). In addition, the results were very similar after additional adjustment for the amount of fluid administered during the clamp and for outpatient dietary salt intake.

## Discussion

In this study of healthy individuals across a wide range of BMIs, we demonstrate that acute exposure to supraphysiologic insulin concentrations led to a modest reduction in circulating NT-proANP levels. Furthermore, lower insulin sensitivity, independent of BMI, was associated with lower NT-proANP concentrations at steady-state during hyperinsulinemic-euglycemic clamp. These observations suggest that insulin action may contribute to the differences in NT-proANP observed in obese compared with lean individuals. The findings underscore the importance of understanding mechanisms by which hyperinsulinemia and/or insulin resistance influence the cardiac hormonal axis.

Our results are consistent with some prior insulin clamp studies [13–15, 18] but not others [11, 12, 16, 17, 19–21]. Pivovarova et al. conducted one of the larger prior studies, in which 25 overweight individuals underwent a hyperinsulinemic-euglycemic clamp and experienced a decrease in circulating midregional-proANP (MR-proANP) levels [13]. In a separate study, the same group found that MR-proANP levels decreased in 14 obese men who underwent hyperinsulinemic clamps [13]. Our study extends these results to a group of individuals across a wider range of BMIs. However, not all prior clamp studies have found a decrease in NP levels in response to insulin [11, 12, 16, 17, 19–21]. The present study addresses some of the limitations of prior studies. First, most prior studies focused on a narrower range of BMIs, making it difficult to know whether observed NP changes were modified by obesity status. Second, most prior insulin clamp studies measured the mature ANP peptide, which is very sensitive to volume status and salt exposure, and undergoes rapid degradation in plasma. Third, very few prior clamp studies measured BNP or BNP propeptides. In the present study, we analyzed the propeptides of both ANP and BNP, which are more stable than the mature peptides. We used improved assays and adjusted for many possible confounders of NP (including outpatient dietary salt intake and the volume received during the infusions). Finally, we reported changes in NP levels from baseline to “steady-state,” defined as the mean NP level at the beginning and end of steady-state, in order to capture the effects on NP levels during the entire steady-state period.

Whether the decreases in NT-proANP in the present study occurred because of decreases in production, or increases in clearance, of NPs is unknown. Exposure of insulin-sensitive mice cardiomyocytes to insulin may stimulate increased BNP expression [23], an effect that may be mediated by phosphatidylinositol 3-kinase-AKT (PI3K-AKT) signaling. The apparent discordance with the human findings could be attributable to differences in NP physiology between rodents and humans, differences in timescale, involvement of NP clearance mechanisms, or activation of alternate intracellular insulin signaling pathways. For instance, insulin administration does appear to stimulate increased expression of the NP clearance receptor in adipose tissue [13], though this is expected to principally influence clearance of mature ANP and BNP, rather than their propeptides. Little is known about the mechanisms by which circulating NP propeptides are cleared. In addition, the differences between the *in vitro* rodent study and the current human study could be related to differential pathway-specific insulin sensitivity. The actions of insulin at the cellular level are complex and involve diverse signaling pathways, including the PI3K-AKT and Mitogen-Activated Protein Kinase (MAPK) pathways [24, 25]. The increase in NP expression in murine cardiomyocytes after exposure to insulin appears to be mediated by the PI3K-AKT pathway [23]. It is possible that insulin affects the NP system via the MAPK pathway [26]. Evidence also exists in other tissues that the PI3K and MAPK pathways can differentially regulate the effects of insulin action [24]. This raises the possibility that the observed decrease in proANP in the human study could potentially reflect the net effect of actions mediated by multiple pathways.

Interestingly, steady-state ANP levels, adjusted for baseline, were lower in individuals with poorer insulin sensitivity, independent of BMI. Thus, although the NT-proANP levels decreased in the group as a whole, individuals who were very sensitive to insulin experienced smaller decreases, or even increases, in NT-proANP levels. These findings are consistent with observations in large cohort studies that patients with insulin resistance (as assessed by HOMA-IR) have lower NP levels independent of BMI [9]. Our study confirms this relationship in a well-characterized group of patients whose insulin sensitivity was measured using the gold standard (glucose disposal rate during a hyperinsulinemic-euglycemic clamp). This study supports the concept that insulin resistance plays a role in the relative “NP deficiency” in obesity. The etiology of NP deficiency in obesity is likely multifactorial and may result from the interactions of several physiologic and pathological factors [10], including insulin resistance, alterations in sex steroids which can affect both body fat distribution and the NP system [10, 27–29], and complex interactions between adipose tissue and cardiac endocrine function [10, 30–40]. Prior studies have supported that substances secreted by adipose tissue, including pro-inflammatory adipokines and some cytokines, may affect NP gene transcription [10, 30–38], and additionally, that NPs play a role in the adipose tissue function and development [10, 39, 40]. These findings suggest that complex interrelationships exist between adipose tissue and the NP system [10] and may play a role in the apparent NP deficiency in obesity. In addition, Obokata recently reported that among obese patients with heart failure with preserved ejection fraction, increased epicardial fat can induce a pericardial restraint that may reduce cardiac wall stress and thus inhibit NP production [41], contributing to a relative NP deficiency in obesity.

In contrast to NT-proANP, NT-proBNP concentrations did not change in response to insulin administration. Consistent with our findings, two prior hyperinsulinemic-euglycemic clamp studies in selected populations did not show an association between insulin infusion and changes in concentrations of BNP or NT-proBNP [13, 20]. We extend these findings to a larger sample of relatively healthy individuals across the BMI spectrum. Differences in the turnover rates between NT-proANP and NT-proBNP may have contributed to the discrepant findings between these two peptides. We measured the propeptides of ANP and BNP because they are more stable in the circulation than the active peptides [42, 43]. However, changes in concentrations of the propeptides may be harder to detect during an acute study, due to the longer half-lives. Furthermore, peptides related to the ANP system have shorter half-lives than peptides related to the BNP system [43]. Thus, it may have been easier to demonstrate the acute effects of insulin with NT-proANP than with NT-proBNP. On the other hand, it is also possible that certain factors may regulate these peptides differentially. We have previously reported that glucose may induce expression of microRNA-425, which regulates production of proANP but not proBNP [44].

Our findings may not apply to chronic changes in NP in response to interventions that impact insulin action or sensitivity. Although NT-proBNP did not change acutely in the present study in response to high-dose insulin administration, changes in NT-proBNP were positively associated with improvements in insulin sensitivity after preventive measures for Type 2 diabetes in the Diabetes Prevention Program, independent of changes in BMI and waist circumference [45].

Moreover, individuals with Type 2 diabetes randomized to pioglitazone experienced improvements in insulin sensitivity in conjunction with increases in NP bioactivity, as determined by the expression of NP receptors in adipose tissue [46]. A challenge of prior studies investigating the chronic effects of insulin sensitivity on NP levels is that interventions that affect insulin sensitivity also often affect glucose levels. Some prior studies suggest that glucose levels may independently affect NP levels [44]. Thus, the hyperinsulinemic-euglycemic clamp technique used in the present study offers the advantage of examining the effects of insulin on NP levels, independent of changes in glucose.

A limitation of the present study is the imbalance in sample size between men and women. Large population studies indicate that circulating NP concentrations are lower in men than women [29, 42, 43]. To address this limitation, we adjusted for sex in multivariable analyses, and also performed analyses in women alone, and found that the major results were unchanged. Similarly, in large population studies, circulating NP levels are significantly lower in obese compared with lean individuals [8, 9, 42]. In the present study, NP levels did not differ significantly between lean, overweight, and obese individuals at baseline, a result that is almost certainly due to chance given the relatively small sample size in each subgroup. Finally, another limitation is that we did not perform routine echocardiography in study participants. Thus, we cannot exclude the possibility that some participants, particularly those with obesity, may have had subclinical diastolic dysfunction.

In conclusion, acute exposure to high-dose insulin led to moderate decreases in circulating NT-proANP, with lower concentrations among more insulin-resistant individuals. Further studies are warranted to elucidate potential mechanisms underlying the effects of insulin on NT-proANP production, and on the chronic effects of insulin on the cardiac hormonal axis.

## Supporting information

S1 Fig Changes in circulating NP concentrations from baseline to the beginning and end of steady-state during hyperinsulinemic-euglycemic clamp. NT-proANP in the group as a whole decreased significantly from baseline to the beginning of steady-state (mean decrease of 1979 ng/L, 95% confidence interval [656–3304], p = 0.005; *Panel A*), and from baseline to the end of steady-state (mean decrease of 1886 ng/L, 95% confidence interval [345–3428], p = 0.03, *Panel B)*. In contrast, NT-proBNP did not change significantly from baseline to the beginning of steady-state *(Panel C)*, or from baseline to the end of steady-state *(Panel D)*.
(TIFF)
