# Supplementary figures and images for: Acute effects of insulin on circulating natriuretic peptide levels in humans
Source: PLoS One. 2018 May 14;13(5):e0196869. doi: 10.1371/journal.pone.0196869 (PMC5951576; doi:10.1371/journal.pone.0196869)

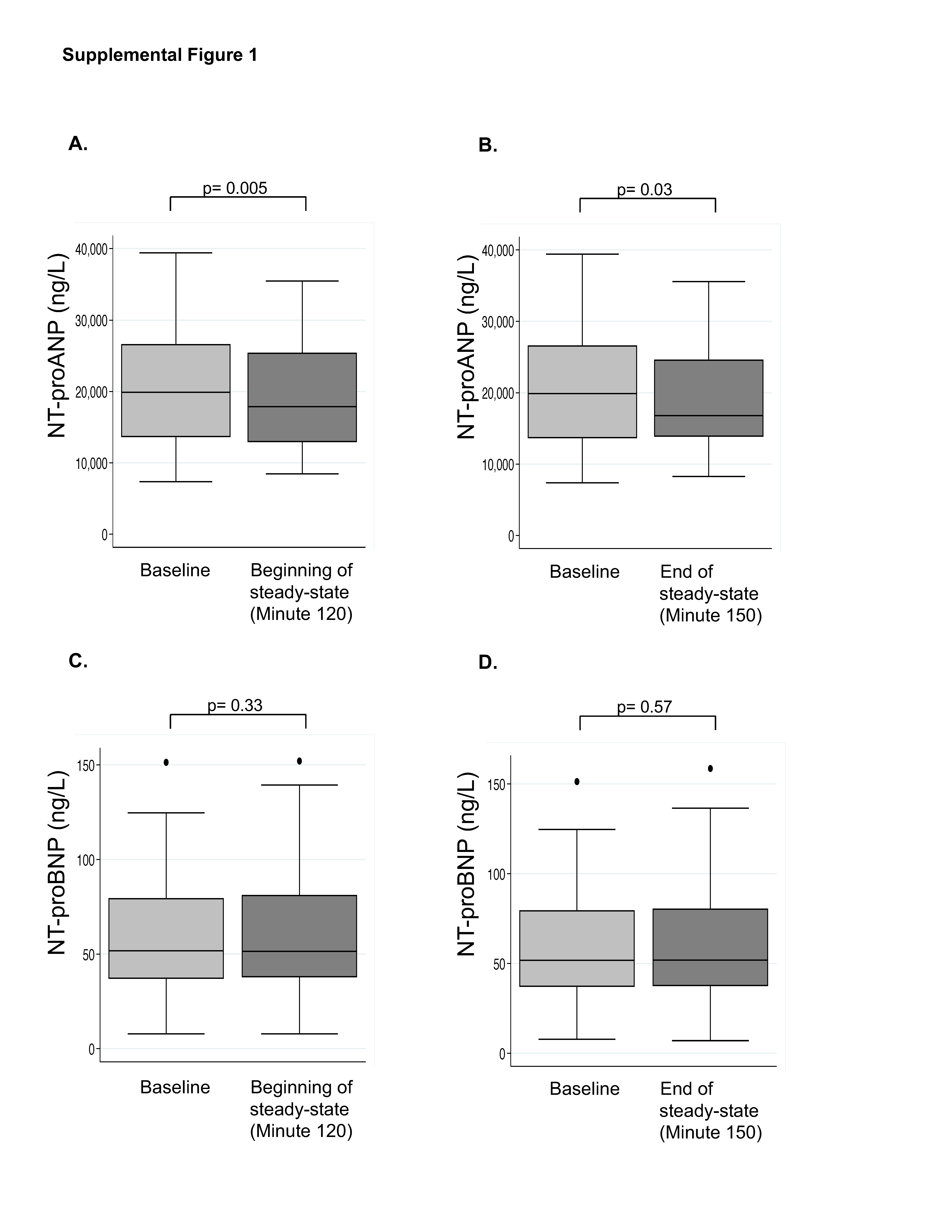

Supplement: S1 Fig — NT-proANP in the group as a whole decreased significantly from baseline to the beginning of steady-state (mean decrease of 1979 ng/L, 95% confidence interval [656–3304], p = 0.005; Panel A), and from baseline to the end of steady-state (mean decrease of 1886 ng/L, 95% confidence interval [345–3428], p = 0.03, Panel B). In contrast, NT-proBNP did not change significantly from baseline to the beginning of steady-state (Panel C), or from baseline to the end of steady-state (Panel D). (TIFF) [file pone.0196869.s001.tiff]
